# Pharyngeal Residue Is Independently Associated With Mortality and Aspiration Events in Patients Undergoing Swallowing Assessment: A Retrospective Cohort Study

**DOI:** 10.1002/jgf2.70148

**Published:** 2026-08-28

**Authors:** Yuki Chiko, Shizuma Omote, Ei Miyasaka

**Affiliations:** ^1^ Department of Internal Medicine Fukuyama Minami Hospital Hiroshima Japan

**Keywords:** aspiration, dysphagia, mortality, pharyngeal residue, swallowing assessment

## Abstract

**Background:**

The prognostic significance of pharyngeal residue during routine swallowing assessment remains unclear in older adults at regional hospitals.

**Methods:**

We conducted a single‐center retrospective cohort study of 107 consecutive patients (median age 87 years) undergoing swallowing assessment at a regional hospital in Japan (January 2021–December 2024; follow‐up until June 2025). The primary outcome was all‐cause mortality, and the secondary outcome was aspiration events. Time‐to‐event analyses used Kaplan–Meier methods, multivariable Cox proportional hazards models, and Fine–Gray subdistribution hazard models to account for the competing risk of death.

**Results:**

During a median follow‐up of 72 days (IQR 36–156), 72 deaths and 63 aspiration events occurred. Pharyngeal residue was independently associated with aspiration events (HR 3.26; 95% CI 1.70–6.26; *p* < 0.001) and all‐cause mortality (HR 2.65; 95% CI 1.38–5.10; *p* = 0.003). These findings were consistent in sensitivity analyses excluding antipsychotic use and in Fine–Gray competing risks analysis (SHR 2.76; 95% CI 1.51–5.04; *p* = 0.001). Dementia was also independently associated with mortality (HR 1.83; 95% CI 1.02–3.29; *p* = 0.044).

**Conclusions:**

Pharyngeal residue was independently associated with all‐cause mortality and aspiration events, supporting its utility as a simple prognostic marker for risk stratification in older adults with suspected dysphagia.

## 1 Introduction

Japan is one of the most rapidly aging societies, and pneumonia continues to impose a substantial clinical burden in older adults. A multicenter prospective study from Japan highlighted the magnitude of community‐onset pneumonia in an aging population, underscoring the relevance of pneumonia as an everyday problem across clinical settings [1]. Among older adults, aspiration‐related mechanisms represent a major contributor to pneumonia, reflecting the high prevalence of dysphagia and impaired airway protection [2].

Importantly, the management of aspiration pneumonia should not be limited to antibiotic treatment alone. Prevention of recurrent aspiration and alignment of care with patients' goals and values—often through shared decision‐making with families regarding feeding strategies—are central to comprehensive care in frail older adults [3] In routine practice, clinicians frequently face the challenge of balancing aspiration risk with quality of life, particularly when swallowing dysfunction is suspected.

Swallowing function assessment is therefore essential to evaluate the safety of oral intake and to guide preventive strategies. Instrumental evaluations such as videofluoroscopic swallowing study (VFSS) and flexible endoscopic evaluation of swallowing (FEES) are widely used to characterize swallowing physiology, including airway invasion and pharyngeal residue. A systematic review and meta‐analysis comparing FEES and VFSS suggested that FEES may have a slight advantage for detecting aspiration, penetration, and residue [4].

Pharyngeal residue reflects impaired bolus clearance, and validated rating tools have been developed to standardize residue assessment, such as the Yale Pharyngeal Residue Severity Rating Scale for FEES [5]; however, the prognostic significance of residue findings in routine practice remains incompletely clarified. In particular, most existing evidence derives from academic or tertiary centers with dedicated swallowing laboratories, and real‐world data from regional hospitals—where resources, evaluation pathways, and specialist expertise are often limited—are scarce.

Therefore, we conducted a retrospective cohort study in a regional hospital to determine whether pharyngeal residue, assessed dichotomously as present or absent from routine swallowing assessment records without specialized scoring systems, is independently associated with all‐cause mortality and subsequent aspiration events. Aspiration events were defined as a clinical diagnosis of aspiration pneumonia with treatment initiation by the attending physician, or a documented episode of overt aspiration or choking requiring suctioning, as ascertained by retrospective chart review. By focusing on an oldest‐old population managed in a regional hospital—an underrepresented setting in prior studies—we aimed to clarify whether this simple and pragmatic swallowing finding may provide clinically meaningful prognostic information for risk stratification and shared decision‐making in routine practice.

## 2 Methods

### 2.1 Study Design and Setting

This was a single‐center retrospective cohort study conducted at Fukuyama Minami Hospital, a regional hospital in Hiroshima, Japan, where swallowing assessments are performed as part of routine clinical care for older adults with suspected dysphagia. Patients were enrolled from January 2021 to December 2024, and follow‐up was continued until June 2025.

### 2.2 Participants

We screened all consecutive patients who underwent swallowing assessment during the study period. For patients who underwent repeated swallowing assessments, only the first assessment was included as the index evaluation. Patients with missing data on pharyngeal residue were excluded from the analysis.

### 2.3 Swallowing Assessment

Swallowing function was evaluated primarily by videofluoroscopic swallowing study (VFSS) in routine clinical practice, while flexible endoscopic evaluation of swallowing (FEES) was performed in a minority of cases when clinically indicated. VFSS or FEES was performed as clinically indicated, typically after the onset of swallowing‐related symptoms or when findings were needed to guide decisions regarding oral intake and treatment strategy, rather than at a fixed time point relative to admission. The number of patients assessed by each modality was as follows: VFSS, *n* = 103; FEES, *n* = 4. Each examination was evaluated jointly by one speech‐language therapist and one physician experienced in swallowing evaluation, who reached a consensus judgment in real time during the examination; this real‐time assessment was subsequently reconfirmed by review of the recorded video, and findings were documented in the medical record. Pharyngeal residue was assessed dichotomously as present or absent, without the use of a formal severity rating scale (e.g., the Yale Pharyngeal Residue Severity Rating Scale or the Normalized Residue Ratio Scale). A standardized assessment protocol was used, including evaluation with food and liquid of varying consistencies (e.g., water, thickened liquid, and jelly), with stepwise progression based on clinical judgment. Assessments were performed with patients in an upright or semi‐recumbent position as determined by the attending speech‐language therapist based on the patient's clinical status; postural adjustments were applied at the discretion of the examiner when clinically indicated. Because both VFSS and FEES allow direct visualization of pharyngeal residue after swallowing, pharyngeal residue was operationally defined identically regardless of modality. Swallowing assessment findings were retrospectively extracted from the medical record for the purposes of this study.

### 2.4 Outcomes and Follow‐Up

The index date was defined as the date of the swallowing assessment. The primary outcome was all‐cause mortality. The secondary outcome was aspiration events, defined as either [1] a clinical diagnosis of aspiration pneumonia by the attending physician with initiation of treatment, or [2] a documented episode of overt aspiration or choking requiring suctioning, as recorded in the medical record. Aspiration events occurring during the swallowing assessment itself were excluded. Outcome ascertainment was performed by retrospective chart review. Follow‐up included post‐discharge outpatient visits and readmissions recorded in the hospital's medical records. Patients without an event were administratively censored at June 30, 2025, or at the date of the last available follow‐up, whichever came first. No objective diagnostic criteria (e.g., radiographic or laboratory findings) were required for the definition of aspiration events; ascertainment was based solely on clinical documentation in the medical record.

### 2.5 Covariates

The following covariates were included in the multivariable analysis based on a priori clinical and biological reasoning informed by a directed acyclic graph (DAG) framework. Age and dementia were selected as potential confounders because the underlying etiology of dysphagia—including dementia‐related, cerebrovascular, and neurodegenerative causes—is a common cause of both pharyngeal residue and adverse outcomes; dementia was used as a single variable to represent the primary dysphagia etiology in this cohort, given the limited event count and the predominance of dementia among the study population. Laryngeal penetration was included as a marker of concurrent airway protective dysfunction. Antipsychotic use was included on the basis that psychoactive medications may affect alertness, swallowing function, and aspiration risk; however, given its inverse distribution between groups—which may reflect reverse causation or a collider structure rather than a pharmacological effect—a sensitivity analysis excluding this variable was pre‐specified and performed (see Results). Antipsychotics were treated as a separate category, whereas psychotropics represented a broader category of psychoactive medications.

### 2.6 Statistical Analysis

Baseline characteristics were compared according to the presence or absence of pharyngeal residue. Time‐to‐event outcomes were analyzed using the Kaplan–Meier method and compared with the log‐rank test. Multivariable Cox proportional hazards models were used to assess the association of pharyngeal residue with aspiration events and all‐cause mortality, adjusting for age, dementia, laryngeal penetration, and antipsychotic use. Hazard ratios (HRs) and 95% confidence intervals (CIs) were reported. A two‐sided *p* value < 0.05 was considered statistically significant. The number of events per variable (EPV) was 14.4 for the mortality model (72 events, 5 variables) and 12.6 for the aspiration model (63 events, 5 variables), both exceeding the conventional threshold of 10. To assess the potential influence of antipsychotic use—which showed an inverse distribution between groups—a pre‐specified sensitivity analysis was performed by excluding antipsychotic use from both Cox models. To account for the competing risk of death on aspiration event ascertainment, Fine–Gray subdistribution hazard models were additionally performed. Complete‐case analysis was performed.

### 2.7 Ethics Statement

This study was approved by Japan Physicians Association Institutional Review Board (approval no. 056–2506‐03). Because this was a retrospective observational study using existing medical records, informed consent was obtained using an opt‐out approach. This study was conducted in accordance with the principles of the Declaration of Helsinki.

Artificial intelligence (AI)–based tools were used in the preparation of this manuscript in the following ways: (1) AI‐assisted language editing tools (including large language model–based assistants) were used to improve the clarity, grammar, and readability of the English text throughout the manuscript, including the Introduction, Methods, Results, Discussion, and Abstract sections; (2) AI assistance was used to support the structuring and drafting of response letters and revision documents during the peer review process. AI tools were not used for data collection, data analysis, statistical modeling, or interpretation of results. All AI‐assisted content was reviewed, verified, and approved by the authors, who take full and sole responsibility for the accuracy, integrity, and final content of this manuscript. The use of AI tools complies with the journal's AI policy as outlined in the Author Guidelines.

## 3 Results

### 3.1 Study Population

The median follow‐up duration was 72 days (interquartile range [IQR], 36–156 days; range, 3–1339 days). During follow‐up, 72 deaths occurred (residue‐present group: 55 [77%]; residue‐absent group: 17 [47%]), and 63 aspiration events were observed (residue‐present group: 50 [70%]; residue‐absent group: 13 [36%]). The remaining patients were censored at the date of last available follow‐up or the administrative censoring date (June 30, 2025), whichever came first.

### 3.2 Baseline Characteristics

**FIGURE 1:**
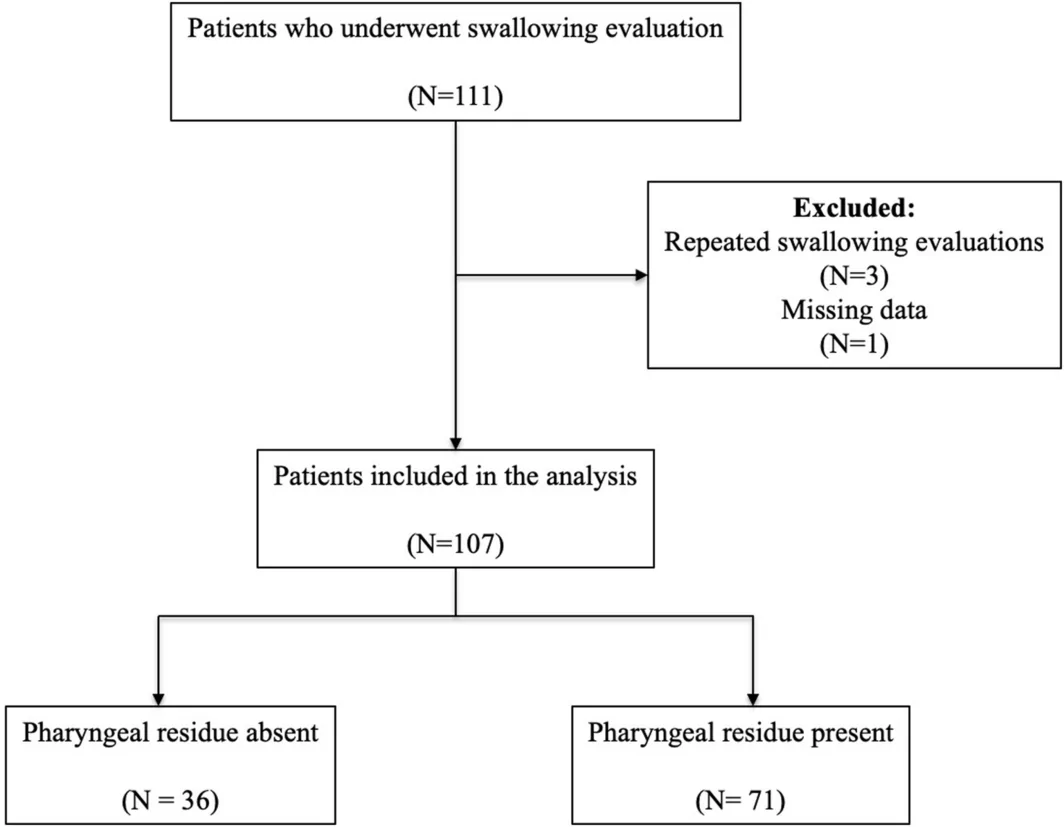
Flow diagram of patient inclusion. A total of 111 swallowing assessments were identified during the study period. After exclusion of repeated swallowing evaluations and patients with missing data on pharyngeal residue, 107 patients were included in the final cohort.

Baseline characteristics according to the presence or absence of pharyngeal residue are shown in Table 1. The flow diagram of patient inclusion is shown in Figure 1. The median age of the overall cohort was 87 years (interquartile range [IQR], 79–92 years), and 40 patients (37%) were female. There were no significant differences between the residue‐absent and residue‐present groups in age, cerebrovascular disease, neurological disease, dementia, diabetes, aspiration history, albumin, or C‐reactive protein levels.

**TABLE 1:** Baseline characteristics according to pharyngeal residue status.

Characteristic	Overall (*n* = 107)	Absent (*n* = 36)	Present (*n* = 71)	*p*
Age, years	87 (79–92)	85 (75–92)	88 (81–92)	0.30
Male sex, *n* (%)	67 (62.6)	18 (50.0)	49 (69.0)	0.055
Cerebrovascular disease, *n* (%)	34 (31.8)	11 (30.6)	23 (32.4)	0.80
Neurological disease, *n* (%)	10 (9.3)	4 (11.1)	6 (8.5)	0.70
Dementia, *n* (%)	74 (69.2)	24 (66.7)	50 (70.4)	0.70
Diabetes mellitus, *n* (%)	18 (16.8)	5 (13.9)	13 (18.3)	0.60
History of aspiration pneumonia, *n* (%)	33 (30.8)	12 (33.3)	21 (29.6)	0.70
Antipsychotic use, *n* (%)	21 (19.6)	11 (30.6)	10 (14.1)	**0.043**
Psychotropic use, *n* (%)	18 (16.8)	9 (25.0)	9 (12.7)	0.11
Albumin, g/dL	2.50 (2.20–2.90)	2.50 (2.30–3.10)	2.50 (2.20–2.80)	0.50
CRP, mg/dL	9 (4–14)	9 (5–12)	10 (4–15)	0.70
Laryngeal penetration, *n* (%)	57 (53.3)	11 (30.6)	46 (64.8)	**< 0.001**
Oral intake resumed, *n* (%)	78 (72.9)	32 (88.9)	46 (64.8)	**0.008**

*Note:* Values are presented as median (interquartile range) or *n* (%). For binary variables, only the presence of each characteristic is shown. *p* values were calculated using the Wilcoxon rank‐sum test, Pearson chi‐square test, or Fisher exact test, as appropriate. Pharyngeal residue was classified as present when residual material remained after additional swallowing. Baseline demographic and clinical characteristics of the study cohort stratified by the presence or absence of pharyngeal residue. Values are presented as median (interquartile range) or number (%), as appropriate. Bold indicate those with a statistically significant difference.

In contrast, antipsychotic use was significantly more frequent in the residue‐absent group than in the residue‐present group (31% vs. 14%, *p* = 0.043). Laryngeal penetration was significantly more common in the residue‐present group than in the residue‐absent group (65% vs. 31%, *p* < 0.001). Resumption of oral intake was less frequent in the residue‐present group than in the residue‐absent group (65% vs. 89%, *p* = 0.008).

### 3.3 Overall Survival

**FIGURE 2:**
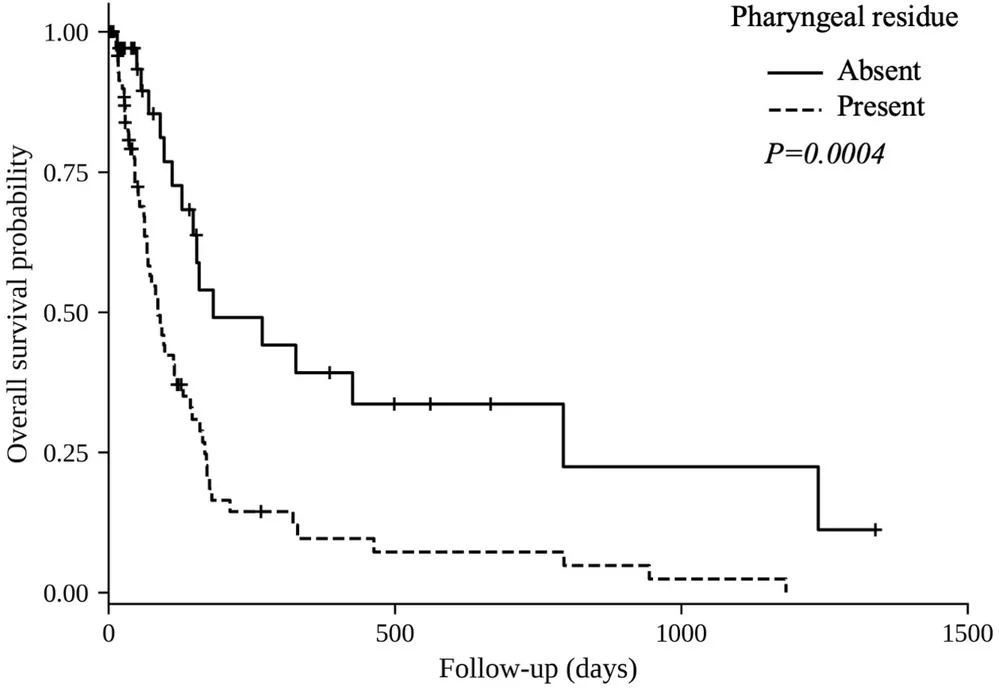
Kaplan–Meier curves for overall survival according to pharyngeal residue. Kaplan–Meier analysis comparing overall survival between patients with and without pharyngeal residue. Patients with pharyngeal residue had significantly lower survival probabilities than those without residue.

Kaplan–Meier analysis demonstrated that patients with pharyngeal residue had significantly lower overall survival than those without (log‐rank *p* = 0.0004) (Figure 2). In the multivariable Cox proportional hazards model adjusted for age, dementia, laryngeal penetration, and antipsychotic use, pharyngeal residue was independently associated with all‐cause mortality (hazard ratio [HR], 2.65; 95% confidence interval [CI], 1.38–5.10; *p* = 0.003). Dementia was also independently associated with mortality (HR, 1.83; 95% CI, 1.02–3.29; *p* = 0.044). Age, laryngeal penetration, and antipsychotic use were not significantly associated with mortality (Table 2). In a sensitivity analysis excluding antipsychotic use from the model, pharyngeal residue remained independently associated with mortality (HR, 2.58; 95% CI, 1.42–4.70; *p* = 0.002), supporting the robustness of the primary finding.

**TABLE 2:** Multivariable Cox proportional hazards analysis for all‐cause mortality (A) and aspiration events (B).

Variable	Primary model		Sensitivity analysis (without antipsychotic)	
	HR (95% CI)	*p*	HR (95% CI)	*p*
A. Mortality (72 events; EPV = 14.4)				
Pharyngeal residue (Yes vs. No)	2.65 (1.38–5.10)	0.003	2.58 (1.42–4.70)	0.002
Age (per year increase)	1.01 (0.98–1.04)	0.414	1.01 (0.98–1.04)	0.432
Dementia (Yes vs. No)	1.83 (1.02–3.29)	0.044	1.76 (0.98–3.17)	0.060
Laryngeal penetration (Yes vs. No)	1.13 (0.68–1.89)	0.627	1.09 (0.66–1.80)	0.736
Antipsychotic use (Yes vs. No)	1.14 (0.55–2.36)	0.715	—	—
B. Aspiration events (63 events; EPV = 12.6)				
Pharyngeal residue (Yes vs. No)	3.26 (1.70–6.26)	< 0.001	3.29 (1.72–6.27)	< 0.001
Age (per year increase)	1.01 (0.98–1.04)	0.510	1.01 (0.98–1.04)	0.511
Dementia (Yes vs. No)	1.46 (0.80–2.66)	0.222	1.44 (0.80–2.62)	0.227
Laryngeal penetration (Yes vs. No)	1.33 (0.78–2.27)	0.292	1.33 (0.78–2.26)	0.296
Antipsychotic use (Yes vs. No)	0.94 (0.47–1.86)	0.853	—	—

*Note:* Primary model: Cox proportional hazards regression adjusted for age, dementia, laryngeal penetration, and antipsychotic use. Sensitivity analysis: same model excluding antipsychotic use. For aspiration events, Fine–Gray subdistribution hazard analysis accounting for the competing risk of death: pharyngeal residue SHR 2.76 (95% CI 1.51–5.04), *p* = 0.001 (primary model); SHR 2.75 (95% CI 1.51–5.03), *p* = 0.001 (sensitivity model). Red text indicates values updated or newly added in the revised manuscript.

Abbreviations: CI, confidence interval; EPV, events per variable; HR, hazard ratio.

### 3.4 Aspiration Events

**FIGURE 3:**
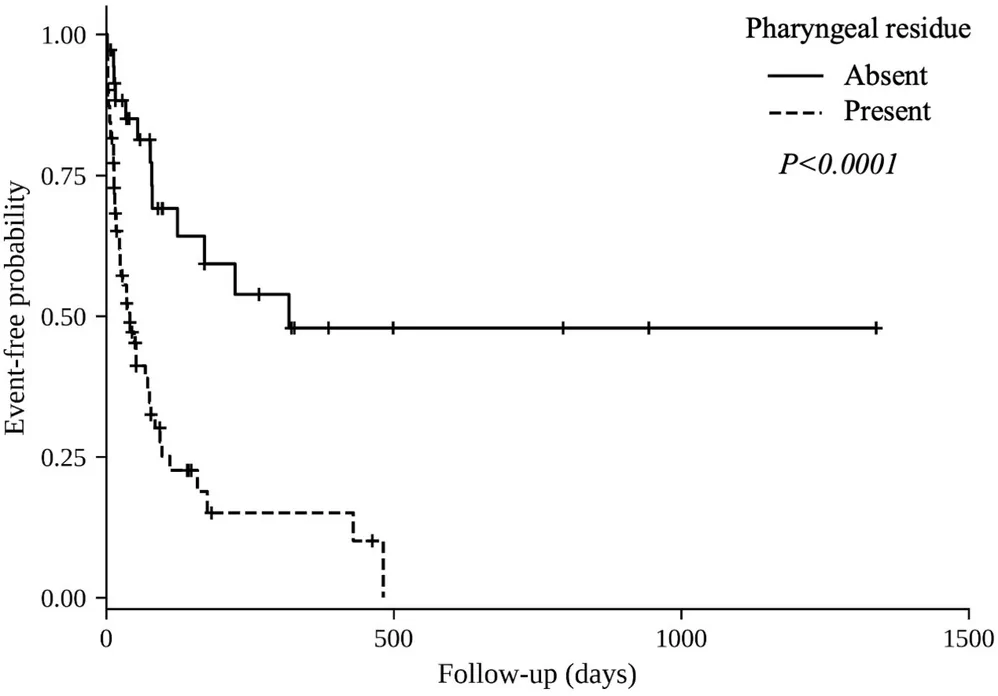
Kaplan–Meier curves for aspiration events according to pharyngeal residue. Kaplan–Meier analysis comparing aspiration‐event–free survival between patients with and without pharyngeal residue. Patients with pharyngeal residue had a significantly higher risk of aspiration events than those without residue.

Kaplan–Meier analysis demonstrated that aspiration events occurred significantly more frequently in patients with pharyngeal residue than in those without pharyngeal residue (log‐rank *p* < 0.0001) (Figure 3). In the multivariable Cox proportional hazards model adjusted for age, dementia, laryngeal penetration, and antipsychotic use, pharyngeal residue was independently associated with aspiration events (hazard ratio [HR], 3.26; 95% confidence interval [CI], 1.70–6.26; *p* < 0.001). None of the other covariates were significantly associated with aspiration events: age (HR, 1.01; 95% CI, 0.98–1.04; *p* = 0.510), dementia (HR, 1.46; 95% CI, 0.80–2.66; *p* = 0.222), laryngeal penetration (HR, 1.33; 95% CI, 0.78–2.27; *p* = 0.292), and antipsychotic use (HR, 0.94; 95% CI, 0.47–1.86; *p* = 0.853) (Table 2). In a sensitivity analysis excluding antipsychotic use, pharyngeal residue remained independently associated with aspiration events (HR, 3.29; 95% CI, 1.72–6.27; *p* < 0.001). To account for the competing risk of death, a Fine–Gray subdistribution hazard model was also performed; pharyngeal residue remained independently associated with aspiration events after accounting for the competing risk of death (subdistribution hazard ratio [SHR], 2.76; 95% CI, 1.51–5.04; *p* = 0.001), and this finding was consistent in the sensitivity model excluding antipsychotic use (SHR, 2.75; 95% CI, 1.51–5.03; *p* = 0.001).

## 4 Discussion

In this single‐center retrospective cohort study of a very elderly population undergoing swallowing assessment in a regional hospital, we found that pharyngeal residue—assessed pragmatically as present or absent in routine clinical documentation—was independently associated with both aspiration events and all‐cause mortality. Specifically, pharyngeal residue was associated with a more than threefold higher risk of aspiration events and a more than twofold higher risk of mortality after adjustment for age, dementia, laryngeal penetration, and antipsychotic use. These findings suggest that pharyngeal residue is not merely a descriptive finding on swallowing assessment but may serve as a clinically meaningful marker of adverse outcomes in older patients with suspected dysphagia. This interpretation is consistent with prior studies indicating that residue‐related swallowing inefficiency is associated with aspiration risk and poor clinical outcomes [6, 7, 8].

Our findings are physiologically plausible. Pharyngeal residue reflects impaired bolus clearance and has long been linked to postdeglutitive overflow aspiration. In the classic videofluoroscopic study by Eisenhuber et al., the risk of aspiration increased markedly with greater residue severity, supporting the concept that retained material in the valleculae or pyriform sinuses can subsequently enter the airway [6]. In another retrospective study of older patients with dysphagia, increased vallecular and pyriform sinus residue was observed more frequently in those with aspiration pneumonia, although pharyngeal delay time and pharyngeal transit time emerged as the strongest independent predictors [7]. Together with our results, these studies support the view that pharyngeal residue may represent not only a marker of abnormal swallowing physiology but also a clinically relevant indicator of increased risk for future aspiration‐related events.

Another important finding in our study is that the association between pharyngeal residue and outcomes remained significant even after adjustment for laryngeal penetration. In our cohort, laryngeal penetration itself was not independently associated with aspiration events or mortality. Although caution is needed when comparing populations, this result is not necessarily inconsistent with prior literature. A recent systematic review in pediatric populations found that the association between laryngeal penetration and aspiration pneumonia appeared weaker and more uncertain than the association for frank tracheal aspiration [9]. This may suggest that pharyngeal residue captures a dimension of swallowing dysfunction that is not fully explained by penetration alone and may therefore provide complementary prognostic information.

The observed association between pharyngeal residue and mortality is also noteworthy. Prior cohort studies have suggested that swallowing assessment findings may have prognostic implications beyond immediate aspiration risk. Nativ‐Zeltzer et al. reported that vallecular residue was independently associated with both incident pneumonia and death within 2 years after VFSS [8]. In a FEES‐based cohort, aspiration detected during endoscopic swallowing evaluation was independently associated with mortality, further supporting the concept that instrumental swallowing findings can reflect overall prognosis in patients with oropharyngeal dysphagia [10]. Our data extend these observations to a regional‐hospital setting in which swallowing evaluations were performed predominantly by VFSS in routine practice and outcomes were captured through retrospective review of medical records, including post‐discharge outpatient follow‐up and readmissions.

These findings may be particularly relevant in real‐world regional practice. In resource‐constrained settings, swallowing assessment is often expected to support not only physiologic description but also clinical decision‐making regarding oral intake, follow‐up intensity, and discussions with patients and families. Our results suggest that the presence of pharyngeal residue may help identify patients at higher risk of subsequent aspiration‐related complications and death, even when other commonly noted findings such as laryngeal penetration are taken into account. From this perspective, careful attention to residue during routine swallowing assessment may contribute to practical risk stratification. This is clinically important because prior work has shown that pneumonia and mortality after instrumental swallowing assessment are not rare outcomes [8, 10].

Several limitations should be acknowledged. First, this was a retrospective single‐center study with a relatively small sample size, raising the possibility of residual confounding and limiting generalizability; although covariate selection was informed by a priori clinical reasoning and a directed acyclic graph (DAG) framework, a formal DAG with an explicit minimal sufficient adjustment set was not constructed, and unmeasured confounding—particularly from the full spectrum of dysphagia etiology (e.g., cerebrovascular, neurodegenerative, sarcopenic, or head‐and‐neck causes)—cannot be excluded. Second, both pharyngeal residue and aspiration events were ascertained from medical record documentation, including post‐discharge outpatient visits and readmissions, without requiring objective diagnostic criteria (e.g., radiographic or laboratory findings) and without formal assessment of inter‐rater reliability; this may have introduced misclassification or under‐ascertainment, particularly for subclinical or silent aspiration, and events occurring outside our institution may not have been fully captured. Third, posture and bolus consistency during swallowing assessment were not systematically standardized or recorded, which may have introduced differential misclassification of pharyngeal residue [11, 12]; this should be addressed in future prospective studies. Fourth, swallowing assessments were performed primarily by VFSS, with FEES used in only a minority of cases (*n* = 4), precluding formal comparison between modalities; although residue was defined identically regardless of modality, differences in detection sensitivity cannot be excluded. Fifth, pharyngeal residue was classified dichotomously as present or absent rather than by a graded severity scale, which may have limited the granularity of prognostic information [6, 8]. Finally, antipsychotic use was more frequent in the residue‐absent group, an inverse distribution that may reflect reverse causation or a collider structure rather than a protective pharmacological effect; this variable was retained in the primary model to avoid omitted‐variable bias, and a sensitivity analysis excluding it confirmed that the primary findings were robust. Because death is a competing event for aspiration ascertainment, we additionally performed Fine–Gray subdistribution hazard analysis, which yielded results consistent with the primary Cox model (SHR, 2.76; 95% CI, 1.51–5.04; *p* = 0.001).

Despite these limitations, our study has important strengths. We focused on clinically meaningful long‐term outcomes—aspiration events and all‐cause mortality—rather than restricting the analysis to findings observed during the swallowing assessment itself. We also included follow‐up beyond discharge, which may better reflect the actual clinical burden experienced by older adults with dysphagia in regional practice. Taken together, our findings support the view that pharyngeal residue is a simple but meaningful marker that may help identify patients at increased risk of adverse outcomes and may inform shared decision‐making in everyday care.

## 5 Conclusion

In conclusion, pharyngeal residue was independently associated with all‐cause mortality and subsequent aspiration events in patients undergoing swallowing assessment in a regional hospital. These findings suggest that pharyngeal residue may serve as a simple and pragmatic marker for risk stratification in older adults with suspected dysphagia. Careful assessment of pharyngeal residue may therefore help inform care planning, follow‐up intensity, and shared decision‐making in routine practice.

## Author Contributions

**Yuki Chiko:** conceptualization, data curation, investigation, validation, formal analysis, project administration, writing – original draft, writing – review and editing. **Shizuma Omote:** supervision, writing – review and editing. **Ei Miyasaka:** supervision, project administration.

## Funding

This research received no specific grant from any funding agency in the public, commercial, or not‐for‐profit sectors.

## Consent

This study used an opt‐out approach for informed consent, as approved by the Japan Physicians Association Institutional Review Board, given its retrospective observational design using existing medical records.

## Conflicts of Interest

The authors declare no conflicts of interest.

## Data Availability

The data that support the findings of this study are available from the corresponding author upon reasonable request.
